# An Evaluation of Prediction Equations for the 6 Minute Walk Test in Healthy European Adults Aged 50-85 Years

**DOI:** 10.1371/journal.pone.0139629

**Published:** 2015-09-29

**Authors:** Michael J. Duncan, Jorge Mota, Joana Carvalho, Alan M. Nevill

**Affiliations:** 1 Centre for Applied Biological and Exercise Science, Coventry University, Coventry, United Kingdom; 2 University of Porto, Faculty of Sports/Research Centre in Physical Activity, Health and Leisure—CIAFEL, Porto, Portugal; 3 Faculty of Health, Education and Wellbeing, University of Wolverhampton, Walsall, United Kingdom; Leeds Beckett University, UNITED KINGDOM

## Abstract

**Objective:**

This study compared actual 6 minute walk test (6MWT) performance with predicted 6MWT using previously validated equations and then determined whether allometric modelling offers a sounder alternative to estimating 6MWT in adults aged 50–80 years.

**Methods:**

We compared actual 6MWT performance against predicted 6MWT in 125 adults aged 50–85 years (62 male, 63 female). In a second sample of 246 adults aged 50–85 years (74 male, 172 female), a new prediction equation for 6MWT performance was developed using allometric modelling. This equation was then cross validated using the same sample that the other prediction equations were compared with.

**Results:**

Significant relationships were evident between 6MWT actual and 6MWT predicted using all of the commonly available prediction equations (all P<0.05 or better) with the exception of the Alameri et al prediction equation (P>0.05). A series of paired t-tests indicated significant differences between 6MWT actual and 6MWT predicted for all available prediction equations (all P<0.05 or better) with the exception of the Iwama et al equation (P = .540). The Iwama et al equation also had similar bias (79.8m) and a coefficient of variation of over 15%. Using sample 2, a log-linear model significantly predicted 6MWT from the log of body mass and height and age (P = 0.001, adjusted R^2^ = .526), predicting 52.6% of the variance in actual 6MWT. When this allometric equation was applied to the original sample, the relationship between 6MWT actual and 6MWT predicted was in excess of values reported for the other previously validated prediction equations (r = .706, P = 0.001). There was a significant difference between actual 6MWT and 6MWT predicted using this new equation (P = 0.001) but the bias, standard deviation of differences and coefficient of variation were all less than for the other equations.

**Conclusions:**

Where actual assessment of the 6MWT is not possible, the allometrically derived equation presented in the current study, offers a viable alternative which has been cross validated and has the least SD of differences and smallest coefficient of variation compared to any of the previously validated equations for the 6MWT.

## Introduction

The six minute walk test (6MWT) is a widely used to asses aerobic endurance in both clinical and non-clinical populations. The 6WT was originally developed to assess the functional capacity of patients with cardiorespiratory diseases [1] but has since been validated in several populations including patients with morbid obesity, Down’s syndrome, Alzheimer’s disease and fibromyalgia amongst others [2,3,4,5,6]. It has also been used as a diagnostic tool in healthy populations from children [7] to older adults [8]. In the case of the latter, the 6MWT features as one of a recommended battery of tests in the senior fitness test [9] to assess functional fitness in older adults.

Although the 6MWT is straightforward and simple to administer there are occasions where such a test cannot be performed (e.g, due to space constraints) or an estimation of 6MWT distance is sufficient. As a consequence a number of authors have developed prediction equations for the 6MWT [10,11,12,13,14,15,16,17,18,19]. The 6MWT is influenced by a number of anthropometric factors with shorter individuals having smaller step length and thus smaller overall 6MWT distances. Fat mass also has a negative effect on 6MWT distance, as does age. It is therefore not surprising that these equations have typically employed measures of height, body mass and age in various combinations to predict 6MWT distance.

The use of prediction equation for the 6MWT may seem attractive for the practitioner, allowing calculation of a metric for exercise performance in a time and labour efficient manner. However, the range of prediction equations available in the literature make it difficult to determine which equations should be used and there are variations in the participant populations such equations were validated upon, ranging from adults over 20 years of age [16], to healthy adults aged 55–75 years old [19]. Despite this, it appears there have been few studies cross-validating these equations. Comparing the efficacy of prediction equations derived on one population with another is an important feature in refining measurement techniques in physiotherapy, exercise science and health. Moreover, all of the existing prediction equations for the 6MWT have been based on multiple linear regression. Such techniques have been criticised in the context of human performance data as lacking a biological basis [20,21,22]. Allometric modelling has been proposed as a suitable technique to overcome this issue due to its sound theoretical basis, biological plausibility and versatile statistical methodology [20,21,22]. To date, no studies appear to have used this approach when modelling performance data in older adults.

The current study sought to address these issues by: a) comparing actual 6MWT distance with 6MWT predicted using commonly used prediction equations in a sample of healthy European adults aged 50 years of age and older; b) using allometric modelling to develop a new prediction equation on an independent sample of healthy European adults aged 50 years of age and older; c) to cross-validate this new equation on the sample of European adults, providing an independent comparison between previously employed prediction equations as the newly created allometric equation.

## Methods

### Study Design

This study employed a three stage design. In stage 1, we compared actual 6MWT performance (sample 1, details below) was compared against 6MWT predicted using all of the previously validated prediction equations in older adults that use height, body mass, sex and age within their models. In Stage 2 we used a separate independent sample (sample 2, details below) to generate a new prediction equation for 6MWT performance using allometric modelling. In Stage 3 we then applied this newly validated equation to our original sample (sample 1), thereby cross validating it on the same sample that the other prediction equations were validated upon, thereby providing a direct comparison between the newly validated and previously validated prediction equations.

### Participants

Two independent samples were recruited in the present study. In both cases, institutional ethics approval, from Coventry University and the University of Porto respectively, and written informed consent were provided prior to any data collection. Both samples consisted of independent, community-dwelling adults aged over 50 years of age. Prior to participation, each participant completed a health history questionnaire to record past and present health conditions. Exclusion criteria were the following: registered blindness, severe hearing impairment uncontrolled hypertension or diabetes, symptomatic cardiorespiratory disease, severe renal or hepatic disease, uncontrolled epilepsy, progressive neurological disease, chronic disabling arthritis or any musculoskeletal condition which prohibited physical activity/exercise. Participants who were currently taking beta blockers of calcium ion channel blockers were also excluded from taking part.

Sample 1 consisted of 125 independent, community-dwelling adults aged 50 years or older (62 male, 63 female, Mean ± S.D. of age = 65.3 ± 7.5 years), recruited from local community groups, within the city of Coventry, UK.

Sample 2 consisted of 246 independent, community-dwelling adults aged 50 years or older (74 male, 172 female, Mean ± S.D. of age = 68.3 ± 5.3 years), recruited from local community groups, within the city of Porto, Portugal.

### Procedures

The procedures used for data collection in both samples were identical and employed the same measurement techniques.

#### Anthropometric Measures

Body mass, measured to the nearest 0.1kg and height, to the nearest 1mm were assessed barefoot with participants wearing light clothing using a Seca Stadiometre and Weighing scales (Seca Instruments, Germany, Ltd).

#### Assessment of Physical Fitness

Physical fitness was assessed using the 6MWT which is commonly used to obtain a measure of submaximal (up to 80% VO_2_ max [23]) physical endurance in older adults [9,23]. The test measures the distance covered when participants are instructed to walk as quickly as they can for 6 minutes. Procedures used followed those recommended by Rikli and Jones [9]. Participants were instructed to walk as quickly as possible for 6 minutes up a down a 20m walkway marked off in 2m segments (cones were used to mark each 2m section), and were informed that they could slow down or rest if necessary. Standardised encouragement was given each minute during the tests. The distance walked was recorded and used for analysis. The protocol employed in the present study does deviate from the originally published protocol by Rikli and Jones [9] where participants are instructed to walk in a 45m rectangular pattern. However, none of the previously validated prediction equations [10,11,12,13,14,15,16,17,18,19] for the 6MWT employed this protocol. Instead, all used straight line shuttle walking. Using straight line walking in the present study therefore enables a better comparison between the present work and previously validated prediction equations.

#### Prediction of Physical Fitness

6MWT performance was then predicted for Sample 1 using eleven previously validated prediction equations [10,11,12,13,14,15,16,17,18,19]. These equations were chosen as they are either recommended for use with participants aged 50 years or more [10, 11, 15, 19] or included participants in excess of 50 years of age in their validation sample [12, 13, 14, 16, 17, 18] and also employ height, body mass and age within a linear model to predict 6MWT performance. For completeness, equations which were validated on non-Caucasian participants including North Africans [13, 14] and Arab populations were also included [17]. Equations which were validated on samples that did not include adults of the age of 50 years or those that employed measures of resting lung function (e.g., [19]) in their prediction were not included.

#### Statistical Analysis

In order to examine the utility of previously validated prediction equations Pearson’s product moment correlations were employed to examine the relationship between 6MWT actual and 6MWT predicted using the various validated regression equations and a series of paired t-tests were then used to examine any differences between 6MWT actual and 6MWT predicted using each of the various validated regression equations. The coefficient of variation was also calculated to provide a measure of the within-subject error variance for each prediction equation. This was performed using the data from sample 1.

Following this, and using independent data from sample 2, multiple regression analysis was used to determine a new prediction equation using a proportional allometric model (equation 1).

6MWT (m)=a X (height)b X (body mass)c X exp(d X age)

After a logarithmic transformation, the allometric model was fitted using ordinary multiple regression to estimate the unknown parameters, a, b, c, and d. This equation, derived from sample 2, was then applied to sample 1 and the aforementioned methods were used to compare this new prediction with actual 6MWT performance. In this way we could develop a new prediction equation on an independent sample (sample 2) and then cross validate it using the same sample (Sample 1) that we used to compare actual 6MWT and predicted 6WMT performance in the first instance (using the previously published 6WMT equations), thus avoiding the statistical phenomenon of shrinkage.

Such methods have been used previously, and recommended, in the context of human performance [21,22,24]. Use of such statistical methods are important when considering either any potential impact of body mass or to compare groups where body mass might differ. Also, the log-linear model approach employed in this study has been shown to be more appropriate than linear models [24] when the performance outcome variable is associated with body size. By using multiple regression, simply by taking logarithms of the dependant variable and entering the logarithmic transformed variables as separate independent variables, the resulting estimated log-linear multiple regression model will automatically provide the most appropriate multiplicative model to reflect the dependent variable based on the proposed allometric model [24]. The statistical package for Social Sciences (SPSS, Version 20) was used for all analysis.

## Results

Results from the first stage of the analysis, using sample 1, indicated significant relationships between 6MWT actual and 6MWT predicted using all of the commonly available prediction equations (all P<0.05 or better) with the exception of the Alameri et al (2009) prediction equation (P>0.05). The strongest of these relationships was the Jenkins et al (2009) equation (r = .673) and the weakest the Alameri et al (2009) equation (r = .083). A series of paired t-tests indicated significant differences between 6MWT actual and 6MWT predicted for all available prediction equations (all P<0.05 or better) with the exception of the Iwama et al [18] equation (P = .540). Despite this, the Iwama et al [18] equation also had considerable bias (79.8m) and a coefficient of variation of over 15%. Pearson’s product moment correlation coefficients between 6MWT actual and 6MWT predicted are presented in Table 1, and Means, Differences (actual—predicted) ± SD and coefficient of variation for 6MWT actual and 6MWT predicted using different validated regression equations is presented in Table 2.

**Table 1:** Pearson’s product moment correlation coefficients between 6MWT Actual and 6MWT Predicted using different validated regression equations.

	6MWT Predicted									
	Enright & Sherill	Gibbons et al., 2001	Troosters et al., 1999	Enright et al., 2003	Camarri et al., 2006	Masmoudi et al., 2008	Alameri et al., 2009	Ben Saad et al., 2009	Iwama et al., 2009	Jenkins et al., 2009
6MWT Actual	.645**	.451**	.589**	.302*	.504**	.582**	.083	.536**	.414**	.673**

*P<0.05.

**P<0.01.

**Table 2:** Mean ± SD and coefficient of variation for 6MWT Actual and 6MWT Predicted using different validated regression equations.

		6MWT Predicted									
	6MWT Actual	Enright & Sherill	Gibbons et al., 2001	Troosters et al., 1999	Enright et al., 2003	Camarri et al., 2006	Masmoudi et al., 2008	Alameri et al., 2009	Ben Saad et al., 2009	Iwama et al., 2009	Jenkins et al., 2009
Means (m)	528.1	512	582	570	489.3	604.3	516.8	507.4	558.6	530.3	573
Differences (Predicted actual)		-31.8	107.9	83.8	-77.5	152	-22.7	-41.4	61	4.4	89.8
SD of Differences		67	78.4	73	69.2	75.7	71.8	88.8	88.4	79.8	67.3
CV(%)		13.1	13.4	12.8	14.1	12.5	13.9	17.5	15.8	15.1	11.7

*P<0.01, significantly different to actual.

In the second stage of the analysis using sample 2, and after log transformation, the log-linear model significantly predicted 6MWT from the log of body mass and height and age (P = 0.001, adjusted R^2^ = .526), predicting 52.6% of the variance in actual 6MWT from the equation:

Distance (male)=(290.6)X (height(cm)0.525)X (body mass (kg)−.317)X exponential (−.009 X age)

Distance (female)=(260.3)X (height(cm)0.525)X(body mass (kg)−.317)X exponential (− .009 X age)

where b = 0.525 (SE = 0.255), c = -0.317 (SE = 0.060) and d = 0.009 (SE = 0.002).

We then took this new prediction equation that was generated on sample 2 and applied it to the original sample (sample 1) thereby evaluating the utility of the new equation with the same sample that we had examined the previously validated equations. When the allometric equation was applied to the original sample, Pearson’s’ product moment correlation was significant (r = .706, P = 0.001), and in excess of values reported for the other previously validated prediction equations. The Paired samples t-test between actual 6MWT and 6MWT predicted using this new equation was significant (P = 0.001). However, the bias with the new equation was less (55.9m) than for the other equations, as was the standard deviation of differences (62.3) and the coefficient of variation (11.2%). Of note, the slope parameter for age in this model was β = 0.009 which was identical to the slope parameter for age on the sample the equation was developed on (sample 2) indicates that a 0.9% reduction in actual 6MWT distance is associated with every year increase in age, and that this was consistent for both sample 1 and 2.

## Discussion

The results of the present study suggest that there is considerable variation between 6MWT actual and 6MWT predicted by the majority of previously validated equations. The correlations between 6MWT actual and 6MWT predicted were not particularly strong, the strongest being r = .673 (Jensen et al., 2009). Only the Iwama et al [18] equation was significantly related to, and also not significantly different from 6MWT actual. These two equations also had considerable bias (SD of differences) and coefficients of variation in excess of 14%. Given the bias between 6MWT actual and 6MWT predicted using any of the previously validated regression equations, we used a two-step approach to create and cross-validate a new allometric prediction equation for the 6MWT using two independent samples. The regression equation developed in this study was more strongly related to actual 6MWT distance and was more accurate than any of the previously validated prediction equations for the 6MWT. The allometric derived equation had smaller standard deviation of differences (62.3) and coefficient of variation (11.2%) than any of the previously validated prediction equations. These improvements in the prediction of 6MWT using the allometric derived model obtained in this study could be considered trivial. It is however important to consider that any improvement in the prediction of a variable is important as it refines the prediction of 6MWT in older adults.

Furthermore, unlike other previously published prediction equations for 6MWT, the current study used a three stage approach where we evaluated the utility of these previously published equations in a sample of European older adults. We then developed an allometric derived prediction equation for the 6MWT on an independent but comparable sample of European older adults. In the third step, we then applied this new equation to our original sample, thereby cross validating the allometric model on the same sample where prior prediction equations had been examined, thus providing a direct comparison between the new and previously validated prediction equations. While there have been a considerable number of equations developed which predict the 6MWT, it is perhaps surprising that few studies have attempted to cross-validate these equations. Without such an analysis, any prediction equation is likely to suffer from the statistical phenomenon of shrinkage. Shrinkage is associated with the quality of fit with a regression model and likely bias any of the previously validated regression equations will be biased as a consequence. This is because as any regression equation will perform best on the sample it was derived from [25]. The approach used in the present study ensures that this is not the case with the two independent samples presented here. It is also important to note that prediction equations are never going to be fully accurate, since motivation, musculoskeletal conditions, cardiopulmonary and metabolic fitness combine in a complex manner to affect performance.

Interestingly, the exponents of height and body mass that were generated as part of the allometric model in Sample 2 were equivalent to Height/Body mass^0.75^. This is in comparison to the typically used Body Mass Index (Height/Body mass^0.5^) in adults and Reciprocal Ponderal Index (RPI: Height/Body mass^0.333^) recommended in children. Although speculative, the changes in ratio between height and mass in the RPI, BMI and with the groups of older adults examined in the current study might be reflective of how the association between body composition and aerobic performance changes with age. Further research is of course required to verify this assertion.

Some caution is also warranted when considering the data presented here. The prediction equations examined in the present study were those that employed simple demographic and anthropometric information (Sex, Height, Body Mass, Age). Other prediction equations are available which include measures of heart rate attained during the 6MWT itself, e.g., [26] or lung function values [19]. These were not included in the present analysis as such data were not available for our sample and inclusion of additional data (e.g. heart rates taken during exercise) would seem to negate the premise on which such regression equations are based, i.e., to provide a valid estimate without the need to physical assessment of a patient. Moreover, the protocol employed in this study deviates from the originally validated protocol of Rikli and Jones [9] where a 45m rectangular track was employed. However, none of the previously validated prediction equations examined here used this approach, preferring instead to adopt a straight line walking/shuttle walk type approach. The approach employed in the present study is therefore consistent with that used by prior authors validating equations for the 6MWT [10,11,12,13,14,15,16,17,18,19]. Of those studies, some employed straight line distances of 20m [16], as did the current study. Others employed 30m [10, 12, 17, 18], 40m [14], 45m [19] and 50m [11] distances in the administration of their tests. Given the practicalities of finding an appropriate indoor area of free space to perform the originally validated Rikli and Jones [9] 45m rectangular protocol, it is perhaps understandable that authors have employed what may be a more practical alternative, in terms of physical space required, in straight line walking. However, these different protocols may result in different patterns of acceleration and deceleration and a different extent to turning demands. The extent to which such differences may impact on overall 6MWT difference is not known but this point is worthy of consideration and the results of the current study should be interpreted bearing this in mind. There are also differences in the level of protocol standardization employed in prior studies with some employing the standardized guidelines recommended by the ATS [1] whereas others, particularly those published prior to 2002, did not. There was however some standardization of protocol in all of the prior studies which validated prediction equations in relation to similarity of verbal encouragement. Standard verbal encouragement tended to be provided every 30s or every minute, depending on the study. It is not known to what extent the variation in encouragement across these different studies may also add to the bias found when comparing the equations they validated with the Caucasian European population examined in the present study.

For completeness, the current study sought to evaluate all of the previously validated 6MWT prediction equations which included adults over the age of 50 years. As a consequence this included studies such as that performed by Iwama et al [18] where the 6MWT was validated in a sample aged 13 years to 84 years, or the equation by Gibbons [16] which is recommended for adults aged 20 years or more. It is therefore important that researchers, scientists and practitioners consider this when deciding which equations might be most suitable for their participant group of interest. Similarly, the participant population examined in the present study was comprised of Caucasian Europeans. Again, in order to provide a comprehensive evaluation of previously validated prediction equations, we also included equations validated on North African [13, 14] and Arab [17] populations. Perhaps unsurprisingly, these performed poorly when comparing predicted to actual 6MWT performance in the current sample. Demographic and anthropometric differences which may exist between individuals of different ethnic backgrounds (e.g., greater height in one ethnicity compared to another) are important considerations when selecting the most appropriate prediction equation to employ. The poor performance of the Masmoudi et al [13] Ben Saad et al [14] and Alameri et al [17] prediction equations serves to highlight how erroneous choice of a prediction equation can result in bias and mistakes in the interpretation of the level of fitness when applied to a sample of different ethnic background compared to the one it was originally validated on.

The use of prediction equations for the 6MWT potentially offers a time and labour efficient way to provide a metric for exercise performance in situations where performance of the actual test may be problematic or not possible. Where actual assessment of the 6MWT is not possible, the allometrically derived equation presented in the current study, offers a viable alternative which has been cross validated and has the least SD of differences and smallest coefficient of variation compared to any of the previously validated equations for the 6MWT. Further research examining the utility of this equation would however be welcome, particularly with non-European or clinical older adult populations.
