# Correction: An Evaluation of Prediction Equations for the 6 Minute Walk Test in Healthy European Adults Aged 50-85 Years

**DOI:** 10.1371/journal.pone.0142463

**Published:** 2015-11-04

**Authors:** Michael J. Duncan, Jorge Mota, Joana Carvalho, Alan M. Nevill

In the Results section, there are errors in both equations. Please view the complete, correct equations here:

$$\text{Distance }\left(\text{male}\right)\text{ = }\left(\text{290.6}\right)\text{ X }\left(\text{height}{\left(\text{cm}\right)}^{\text{0.525}}\right)\text{ X }\left(\text{body mass }{\left(\text{kg}\right)}^{-\text{.317}}\right)\text{ X exponential }\left(-\text{ .009 X age}\right)$$

$$\text{Distance }\left(\text{female}\right)\text{ = }\left(\text{260.3}\right)\text{ X }\left(\text{height}{\left(\text{cm}\right)}^{\text{0.525}}\right)\text{ X }\left(\text{body mass }{\left(\text{kg}\right)}^{-\text{.317}}\right)\text{ X exponential }\left(-\text{ .009X age}\right)$$
